# Good overall behavioural adjustment in children and adolescents with classic congenital adrenal hyperplasia

**DOI:** 10.1007/s12020-020-02244-1

**Published:** 2020-03-09

**Authors:** Valeria Messina, Tatja Hirvikoski, Leif Karlsson, Sophia Vissani, Lena Wallensteen, Rita Ortolano, Antonio Balsamo, Anna Nordenström, Svetlana Lajic

**Affiliations:** 1 Department of Women’s and Children’s Health, Karolinska Institutet, Pediatric Endocrinology Unit (QB83), Karolinska University Hospital, SE-17176 Stockholm, Sweden; 2 Department of Women’s and Children’s Health, Pediatric Neuropsychiatry Unit, Center for Neurodevelopmental Disorders at Karolinska Institutet (KIND), Karolinska Institutet, SE-17177 Stockholm, Sweden; 3 Unit for Habilitation & Health, Stockholm County Council, Stockholm, Sweden; 4 Department of Medical and Surgical Sciences, S. Orsola-Malpighi University Hospital, Pediatric Endocrinology Unit, 40138 Bologna, Italy

**Keywords:** Behaviour, Cognition, Congenital adrenal hyperplasia, Glucocorticoid, Dexamethasone, Prenatal treatment

## Abstract

**Purpose:**

Patients with classic congenital adrenal hyperplasia (CAH) are treated postnatally with life-long glucocorticoid (GC) replacement therapy. Although prolonged exposure to GCs may have a negative impact on behaviour, few studies have studied this issue. We therefore investigated behavioural outcomes in male and female children and adolescents with CAH.

**Methods:**

An observational study in which Swedish and Italian children and adolescents with CAH identified through neonatal screening for CAH (*n* = 57, age range 7–17 years) were compared with healthy population controls matched for age and sex (*n* = 72, age range 7–17 years). Thirteen (eight females) of the fifty-seven children and adolescents with CAH had been treated prenatally with dexamethasone (DEX). Standardised questionnaires for parents and self-report scales for children/adolescents were used to assess behavioural and emotional problems, social anxiety, temperament and scholastic competence.

**Results:**

There were no statistically significant differences between CAH patients (not prenatally treated with DEX) and controls on most of the scales measuring adaptive functioning or behavioural problems. However, children with CAH were rated by their parents to have more social problems than controls (Child Behaviour Checklist, CBCL social problems, *p* = 0.032). In the small group (*n* = 13) of prenatally DEX-treated cases parents rated their children/adolescents to have more mood problems compared with non-DEX-treated children/adolescents with CAH (CBCL-withdrawn/depressed, *p* = 0.019).

**Conclusion:**

Children/adolescents with CAH showed good overall adjustment. The clinical significance of the parentally perceived increase in social problems in children/adolescents with CAH requires further investigation. The findings underline the importance of psychological support for children/adolescents with a chronic condition.

## Introduction

Congenital adrenal hyperplasia (CAH) is a group of autosomal recessive disorder. The most common variant, caused by 21-hydroxylase deficiency, occurs in 1:10,000–1:15,000 live births. CAH is characterised by impaired synthesis of cortisol and aldosterone in the adrenal cortex with a consequent increase in adrenal androgens [1, 2]. In the more severe classic CAH (salt-wasting, SW and simple virilising, SV) the increased production of androgens leads to virilisation of external genitalia in affected girls already in utero [3, 4].

Neonatal screening programmes for CAH have been instituted in Sweden and in many other countries worldwide to facilitate early diagnosis and prevent salt-losing crises and death in neonates [5–7]. Consequently, life-long replacement therapy with hydrocortisone (HC) and mineralocorticoid is already started in the second week of life [1, 8]. However, because of difficulties in mimicking the normal circadian cortisol rhythm with conventional glucocorticoid (GC) treatment, there is a risk of both over- and under-treatment during an individual’s lifespan. A dysregulated or flattened rhythm may be related to behavioural changes and mental health symptoms in children during childhood and early adolescence [9–12].

GCs act via the mineralocorticoid receptor and glucocorticoid receptor. These receptors are highly expressed in the prefrontal cortex, which is linked to regions of the brain (e.g. amygdala, hippocampus and cerebellum) involved in emotional behaviour and stress responses [13, 14]. A few studies investigating structural brain differences in adolescents and adult patients with CAH have reported white matter impairment in the corpus callosum, cerebellum and amygdala, regions that play an important role in emotional regulation. These studies have also reported abnormalities in the hippocampus, a brain region also involved in the regulation of stress and the hypothalamic–pituitary–adrenal axis [15, 16]. A functional magnetic resonance imaging study of the brain reported altered amygdala function in response to negative stimuli (angry and fearful facial expression) in girls with CAH, suggesting impairments in social cognition that may negatively affect social interactions [17].

Studies on behavioural outcomes in children and adolescents with CAH are scarce but the few studies that exist have focused on the effects of prenatal androgen exposure on behaviour in girls and women with CAH. Females with CAH spend more time in male-typical activities in adolescence [18, 19]. Moreover, they are reported by their mothers to be more aggressive and active than their unaffected sisters [20]. These studies support the view that prenatal androgen exposure has a direct organisational effect on the human brain to determine certain aspects of sex-typed behaviour. The majority of girls with CAH, however, have a female-typical gender identity [21].

In addition, epidemiological studies have shown that girls and women with CAH [22], but also men with CAH [23], have substance misuse, mood and anxiety disorders and an increased risk of psychiatric disorders. An increased rate of suicide or suicide attempts has also been reported in men with CAH [24].

Depression [25] and anxiety [26] have been reported in children and adolescents with Cushing’s syndrome, a disease characterised by prolonged exposure to elevated levels of GCs [27]. Of note, the incidence of mood-related problems in patients with Cushing’s syndrome seems to correlate with the degree and timing of GC exposure [28]. Patients with Cushing’s syndrome showed significant improvement in depression and anxiety scores after a reduction of cortisol levels.

Another aspect of the clinical management of CAH concerns prenatal treatment. Since the mid-1980s, in many medical centres worldwide prenatal treatment of CAH with dexamethasone (DEX) has been practised to reduce the in utero virilisation in affected girls. Although effective, the treatment is still controversial because of uncertainties regarding its long-term safety [29–31]. In our previous work on a cohort of DEX-treated children and adolescents in the age range of 7–17 years (with and without CAH) no differences were found in parent-reported behavioural problems compared with population controls. However, DEX-treated children and adolescents scored themselves as having more social anxiety [32, 33]. Conversely, we found that healthy children and adolescents treated during the first trimester with DEX were well adjusted [34].

Living with a child with a chronic condition may also affect parenting behaviour and the parent–child relationship, both of which play an important role in the psychological development of the child. Parental overprotection has been related to poor child adjustment and negative health outcomes (decrease in autonomy, depression and anxiety) [35–37]. Studies on women with CAH show that the disease had an influence on their upbringing with a feeling of being overprotected by their parents and that it may affect their schooling because of frequent hospital visits and the inability to attend physical education classes [38].

Thus, the primary purpose of this study was to assess the behavioural outcomes in a cohort of girls and boys with classic CAH who were diagnosed through national neonatal screening programmes for CAH in Sweden and Italy. In addition, we sought to determine some behavioural outcomes in a small cohort of children and adolescents with CAH who were prenatally treated with DEX.

## Methods

### Recruitment and participants

This study is part of a longitudinal study investigating short- and long-term effects of prenatal treatment with DEX in fetuses at risk of CAH and effects of postnatal chronic GC treatment in patients with CAH (PREDEX study) [31, 34, 39–41]. In this study we include patients from both Sweden and Italy. We focus on the behavioural outcome of children and adolescents with classic CAH and also observe a small group of patients with CAH who were treated prenatally with DEX (boys during the first trimester and girls during the entire gestational period).

All individuals were initially contacted by letter along with information about the study. All those willing to participate gave their written informed consent to take part in the study. Swedish patients were recruited throughout Sweden to minimise selection bias; healthy controls were enlisted from Stockholm County through the Swedish population registry. All the Swedish participants received reimbursement of 50 euros. Italian patients were recruited from the Emilia-Romagna region and the controls from a group of healthy siblings to patients with CAH. All patients and controls were matched for sex and age. The study was approved by the Regional Ethics Committee in Stockholm and the Ethic Committee of the University of Bologna. The participation rate was 81% for the CAH cohort and 55% for the controls.

In total, the CAH cohort comprised 44 children and adolescents with CAH not prenatally treated with DEX (CAH: 22 boys (seven Italian), 22 girls (five Italian)) and 13 children/adolescents with CAH prenatally treated with DEX (CAH-DEX: five boys and eight girls (two Italian)). The control group included 72 individuals (34 boys (one Italian), 38 girls (one Italian); of these, eight were healthy siblings of five patients with CAH included in the study). All participants were between 7 and 17 years old.

All patients with CAH were identified through the Swedish or Italian neonatal screening programmes for CAH (30 had SW CAH and 14 SV CAH). Among the patients with CAH, 14 had a null genotype (completely abolished 21-hydroxylase activity). All patients were treated with HC.

Group-specific demographic data are presented in Table 1. Age and parental education did not differ between the CAH, CAH-DEX and control groups. We show length in centimetres (cm) and birth weight in grams (g), adjusted for country of origin. Moreover, length and weight at birth were converted into *z*-scores according to the Swedish and Italian population-based longitudinal reference values [42, 43]. Children and adolescents with CAH (without prenatal treatment) were longer at birth than population controls (*p* = 0.022); girls treated with DEX during the entire gestational period were born significantly smaller (*p* = 0.019) than CAH girls not treated prenatally (Table 1).

**Table 1:** Demographic data for the three study groups: patients with CAH not treated prenatally with DEX (CAH), patients with CAH treated prenatally with DEX (CAH-DEX) and population controls (C)

	Females			Males			*p* (CAH) CAH vs. C	*p* (DEX) CAH-DEX vs. CAH (f)	*p* (DEX) CAH-DEX vs. CAH (m)
	CAH	CAH-DEX	C	CAH	CAH-DEX	C			
Number of participants	22^a^	8^b^	38^c^	22^d^	5	34^e^			
Age (Mean (SD))	10.8 (3.1)	11.1 (3.5)	10.7 (2.8)	12.3 (3.0)	11.5 (0.9)	10.7 (2.2)	0.099	0.852	0.561
Parental higher education (%)^f^	33.3	42.9	64.9	70.6	40.0	62.5	0.218	0.647	0.218
Birth length^g^ (cm) Mean (SD)	50.6 (3.5)	46.7 (4.0)	49.1 (3.1)	51.5 (2.1)	51.6 (3.2)	50.1 (2.8)	**0.022**	**0.016**	0.949
Birth weight^h^ (g) Mean (SD)	3496.0 (733.0)	2743.7 (757.0)	3245.2 (616.7)	3606.9 (399.1)	3663.0 (752.0)	3488.2 (678.1)	0.092	**0.019**	0.988
Birth length (z-score)	0.6 (1.9)	−1–1 (1.5)	−0.2 (1.3)	0.4 (1.3)	−0.2 (1.6)	−0.4 (1.5)	**0.010**	**0.030**	0.410
Birth weight (z-score)	0.3 (0.6)	−0.4 (0.7)	−0.2 (0.6)	0.1 (0.6)	−0.1 (0.5)	0.0 (0.7)	**0.019**	**0.015**	0.562
Gestational week (SD)	39.1 (2.2)	37.5 (2.6)	39.1 (2.2)	39.7 (2.0)	40.3 (1.1)	39.7 (2.0)	0.505	0.300	0.322
HC (mg/m^2^) (Mean (SD))	12.5 (3.9)	11.5 (4.0)	NA	13.6 (4.7)	14.6 (2.5)	NA		0.576	0.531

Significant *p* values are marked in bold

*HC* hydrocortisone dose, *SD* standard deviation, *N/A* not applicable

^a^Five Italian

^b^Two Italian

^c^One Italian

^d^Seven Italian

^e^One Italian

^f^Education at the university level (%)

^g,h^Values adjusted for country of origin. Statistical analyses for differences between groups were calculated using Fisher’s exact test (two-sided)

### Outcome measures

The questionnaires were completed by the parents and their children when visiting the clinical psychologists. Consequently, they had the opportunity to ask for clarification of items, as recommended in the manual [44].

#### Parent-completed questionnaires

Data to assess behavioural problems, social phobia and temperament were obtained using three parental ratings: The Child Behaviour Checklist for ages 4–18 years (CBCL/4–18), the Social Phobia and Anxiety Inventory for Children—Parent Report (SPAI-C-P) and the Emotionality–Activity–Sociability–Shyness Temperament Survey for children (EAS).

The CBCL/4–18 is an 113-item standardised parental–child instrument for psychiatric screening providing both broadband and narrowband scales. It was used to quantify not only internalising and externalising problems, as well as specific subscales, such as depressive, social and attention problems, but also delinquent and aggressive behaviours [44]. The CBCL total competence score, which measures adaptive functioning, is a sum of scores from three subscales: (1) Activities scale—the number, amount and quality of the child’s participation in different leisure activities and everyday tasks; (2) Social scale—participation in social activities, number of close friends and weekly contact with them; and (3) School scale—(e.g. academic performance and need for a special class or support) [44].

The SPAI-C-P is a 26-item parent-report measure covering cognitive, physiological and behavioural symptoms of social phobia according to the DSM-IV classification system [45, 46]. The SPAI-C-P includes subscales for ‘Public Performance’, ‘Assertiveness/General Conversation’ and ‘Traditional Social Encounters’.

Temperament was quantified using the parent-reported EAS with subscales describing four dimensions of temperament: Sociability, Activity, Shyness and Emotionality [47].

#### Child-completed questionnaires

The Social Anxiety Scale for Children Revised (SASC-R), a self-reported scale for children, was used to assess children’s self-perception of social anxiety and avoidance [48, 49]. The scale is divided into three subscales: Fear of Negative Evaluation (SASC-FNE)—fear of negative evaluation from peers; Social Avoidance and Distress in new social situations or with unfamiliar peers (SAD-New); and Social Avoidance and Distress in general situations (SAD-General).

Children and adolescents also estimated their scholastic ability by completing the Scholastic Competence subscale from the Self-Perception Profile for Children [50]. The questionnaire includes school-related items reflecting the children’s perception of their competence at school.

### Statistical analysis

Multiple linear regression analyses were applied to analyse the role of diagnostic status (CAH, CAH-DEX, control), sex, age, parental education and city of origin on behavioural outcomes (dependent variables). The alpha level was set at *p* < 0.05. Interactions (*p* < 0.10) between sex and group (CAH, control) were followed up by separate post hoc analyses between patients and controls of the same sex. Effect sizes were calculated using Cohen’s *d* based on group differences adjusted for parental education and country of origin in the linear model. In the model a positive effect size represents higher scores in the CAH cohort and negative values represent higher scores in the controls. Effect sizes were categorised as large (*d* ≥ 0.80), moderate (*d* ≥ 0.50) and small (*d* ≥ 0.20). We did not control for multiple comparisons in order not to miss small, though potentially clinically relevant, effects.

Two multiple regression analyses were performed to determine the effect of genotype (null vs. non-null) and phenotype (SW vs. SV) on social problems within the CAH group. To investigate which factors contributed to an increased risk of social problems we included sex, age and GC dose at the time of testing in both of these regression analyses.

Finally, we performed a multiple linear regression analysis to compare behavioural outcomes in children/adolescents with CAH who were prenatally treated with DEX with those with CAH not exposed to DEX in utero. SPSS version 24 (IBM, Armonk, NY, USA) was used for statistical analysis.

## Results

Here, we present adaptive functioning, behavioural and temperamental outcomes for children and adolescents with CAH treated with HC from birth and the impact of prenatal DEX treatment. Group averages for all completed questionnaires, regression coefficients (β), *p* values for the main effects of CAH, CAH by sex interactions as well as effect sizes are summarised in Table 2; the main effect of DEX, DEX by sex interactions as well as effect sizes are summarised in Table 3.

**Table 2:** Results of parent-completed and children’s self-report questionnaires showing group means and standard deviations for patients with CAH not prenatally treated with DEX (CAH) and population controls (C)

	Females			Males			CAH			CAH × SEX	
	CAH *n* = 22	C *n* = 38	Cohen’s *d*	CAH *n* = 22	C *n* = 34	Cohen’s *d*	*β* (CI lower. CI upper)	*p*	Cohen’s *d*	*β* (CI lower. CI upper)	*p*
CBC^a^											
Total competence scale	17.6 (4.3)	16.9 (3.0)	0.26	17.7 (3.8)	17.1 (2.8)	0.25	0.3 (−2.4, 6.0)	0.400	0.26	−0.2 (−3.4, 2.0)	0.598
Withdrawn/Depressed	1.6 (1.5)	1.1 (1.1)	0.50	1.4 (1.7)	1.7 (2.1)	−0.23	0.1 (−1.6, 2.6)	0.627	0.08	−0.3 (−1.8, 0.9)	0.495
Somatic complaints	1.2 (1.5)	1.1 (1.4)	0.05	1.0 (1.1)	0.9 (1.4)	0.12	−0.1 (−2.2, 1.4)	0.644	0.07	0.2 (−0.9, 1.4)	0.647
Anxious/Depressed	4.9 (5.1)	3.4 (3.5)	0.50	2.0 (2.6)	2.9 (4.1)	−0.39	0.3 (−2.5, 7.5)	0.316	0.08	−0.5 (−4.9, 1.5)	0.292
Social problems	2.1 (2.2)	0.7 (1.4)	1.05	0.9 (1.0)	1.5 (2.5)	**−0.50**	0.6 (0.1, 4.6)	**0.042**	0.26	−0.8 (−3.0, −0.1)	**0.032**
Thought problems	0.9 (1.7)	0.3 (0.6)	0.63	0.5 (1.1)	0.9 (2.6)	−0.30	0.2 (−1.0, 2.5)	0.441	0.06	−0.4 (−1.8, 0.4)	0.213
Attention problems	2.7 (3.2)	1.6 (2.1)	0.57	1.7 (2.5)	3.3 (3.9)	−0.69	0.3 (−1.6, 5.6)	0.266	−0.11	−0.7 (−4.2, 0.3)	0.095
Delinquent behaviour	1.8 (3.2)	0.7 (1.1)	0.64	1.2 (1.6)	1.0 (1.5)	0.18	0.4 (−0.8, 3.7)	0.193	0.46	−0.5 (−2.2, 0.6)	0.267
Aggressive problems	3.9 (4.7)	4.8 (4.6)	−0.27	4.1 (5.4)	5.3 (5.2)	−0.32	−0.2 (−8.6, 4.2)	0.505	−0.29	0.1 (−3.4, 4.8)	0.742
Internalising problems	7.5 (7.0)	5.5 (4.3)	0.50	4.5 (3.9)	5.9 (6.9)	−0.35	0.2 (−4.7, 9.6)	0.498	0.06	−0.3 (−6.4, 2.7)	0.414
Externalising problems	5.1 (5.7)	5.5 (5.4)	−0.12	5.3 (6.4)	6.5 (6.6	−0.26	−0.1 (−9.5, 6.0)	0.666	−0.19	0.0 (−4.6, 5.2)	0.907
Total problems	25.9 (21.2)	15.9 (13.1)	0.80	16.5 (12.8)	19.5 (19.6)	−0.26	0.4 (−6.0, 36.5)	0.157	0.28	−0.6 (−23.0, 4.2)	0.177
SPAI-C-P^b^											
Assertiveness/General conversation (AGC)	3.8 (3.2)	4.0 (2.6)	−0.10	2.6 (2.4)	3.5 (3.0)	−0.50	−0.0 (−3.8, 3.6)	0.954	−0.30	−0.1 (−2.7, 2.0)	0.778
Traditional social encounters (TSE)	0.9 (1.5)	1.0 (1.2)	−0.04	0.5 (1.0)	0.9 (1.4)	−0.49	−0.0 (−1.6, 1.7)	0.941	−0.26	−0.2 (−1.4, 0.7)	0.556
Public performance (PP)	4.7 (3.0)	3.1 (2.8)	0.79	2.2 (2.4)	2.3 (2.7)	−0.06	0.4 (−1.4, 5.5)	0.213	0.35	−0.5 (−3.5, 0.9)	0.232
Total	10.2 (8.1)	9.0 (6.3)	0.23	5.9 (5.4)	7.7 (6.9)	−0.43	0.1 (−7.1, 10.3)	0.717	−0.09	−0.3 (−7.4, 3.6)	0.500
EAS^c^											
Emotionality	2.6 (0.9)	2.9 (0.8)	−0.43	2.4 (0.8)	2.6 (1.0)	−0.30	−0.2 (−1.5, 0.7)	0.484	−0.37	0.2 (−0.5, 0.9)	0.627
Activity	3.1 (0.7)	3.4 (0.7)	−0.53	3.0 (0.7)	3.4 (0.7)	−0.82	0.0 (−0.8, 0.9)	0.876	−0.67	−0.2 (−0.7, 0.4)	0.598
Shyness	2.4 (0.8)	2.2 (0.8)	0.30	2.0 (0.9)	1.9 (0.9)	0.11	0.2 (−0.8, 1.4)	0.567	0.19	−0.3 (−1.0, 0.5)	0.536
Sociability	3.3 (1.0)	3.5 (0.8)	−0.38	3.3 (1.0)	3.4 (0.7)	−0.11	−0.0 (−0.9, 0.8)	0.887	−0.25	0.2 (−0.4, 0.7)	0.597
SASC-R^d^											
Fear of negative evaluation (FNE)	14.2 (5.1)	14.8 (5.2)	−0.15	13.0 (5.5)	15.7 (7.7)	−0.57	0.1 (−7.3, 9.2)	0.823	−0.38	−0.3 (−7.0, 3.5)	0.509
Social avoidance and distress (SAD) New	14.0 (3.9)	13.3 (4.3)	0.24	11.6 (3.4)	13.7 (6.0)	−0.61	0.3 (−3.6, 8.7)	0.408	−0.21	−0.5 (−6.0, 1.9)	0.302
Social avoidance and distress (SAD) General	7.4 (2.9)	6.8 (2.4)	0.32	5.8 (1.6)	7.7 (3.7)	−0.97	0.4 (−1.6, 5.9)	0.258	−0.32	−0.7 (−4.2, 0.5)	0.127
Total	35.6 (9.3)	34.8 (9.6)	0.12	30.4 (8.5)	37.2 (14.6)	−0.80	0.2 (−9.2, 20.5)	0.450	−0.38	−0.5 (−15.1, 3.7)	0.237
Self-Perception Profile for Children											
Total school competence	3.1 (0.6)	3.4 (0.4)	−0.63	3.3 (0.4)	3.3 (0.4)	0.15	−0.5 (−1.0, 0.2)	0.150	−0.28	0.6 (−0.1, 0.6)	0.212

Regression coefficients (β) with 95% confidence intervals (CI lower, CI upper), *p* values for the main effect of CAH and interaction between CAH and SEX and effect size (Cohen’s *d*) adjusted for parental education and country of origin are listed for all findings. Significant *p* values are marked in bold

^a^Child Behaviour Checklist (CBCL)

^b^Social Phobia and Anxiety Inventory for Children—Parent Report (SPAI-C-P)

^c^Emotionality–Activity–Sociability–Shyness Temperament Survey for children (EAS)

^d^Social Anxiety Scale for Children Revised (SASC-R)

**Table 3:** Results of parent-completed and children’s self-report questionnaires showing group means and standard deviations for patients with CAH not prenatally treated with DEX (CAH) and patients with CAH prenatally treated with DEX (CAH-DEX)

	Female			Male			DEX			DEX × SEX	
	CAH-DEX *n* = 8	CAH *n* = 22	*d*	CAH-DEX *n* = 5	CAH *n* = 22	*d*	*β* (CI lower. CI upper)	*p*	*d*	*β* (CI lower. CI upper)	*p*
CBCL^a^											
Total competence scale	14.2 (5.2)	17.6 (4.3)	−1.02	14.1 (2.9)	17.7 (3.8)	−1.49	−0.5 (−13.2, 3.7)	0.263	−1.20	0.2 (−4.5, 7.0)	0.660
Withdrawn/Depressed	3.1 (2.5)	1.6 (1.5)	1.04	0.4 (0.5)	1.4 (1.7)	−1.12	1.1 (1.0, 8.5)	**0.014**	0.40	−1.4 (−5.6, −0.5)	**0.019**
Somatic complaints	2.1 (1.5)	1.2 (1.5)	0.91	1.2 (1.3)	1.0 (1.1)	0.18	0.5 (−1.3, 4.7)	0.253	0.69	−0.5 (−2.8, 1.2)	0.428
Anxious/Depressed	2.9 (2.5)	4.9 (5.1)	−0.72	2.4 (2.3)	2.0 (2.6)	0.26	−0.4 (−11.8, 4.6)	0.379	−0.29	0.4 (−3.7, 7.4)	0.506
Social problems	1.3 (2.2)	2.1 (2.2)	−0.54	2.6 (1.7)	0.9 (1.0)	1.79	−0.7 (−6.7, 0.5)	0.093	0.23	1.1 (0.1, 5.0)	**0.047**
Thought problems	1.3 (1.9)	0.9 (1.7)	0.32	0.2 (0.4)	0.5 (1.1)	−0.42	0.3 (−1.5, 3.3)	0.435	0.19	−0.2 (−2.0, 1.4)	0.703
Attention problems	3.6 (4.1)	2.7 (3.2)	0.35	2.6 (3.2)	1.7 (2.5)	0.43	0.2 (−4.9, 8.0)	0.626	0.45	−0.1 (−4.6, 4.1)	0.901
Delinquent behaviour	1.4 (1.6)	1.8 (3.2)	−0.21	3.0 (4.6)	1.2 (1.6)	0.72	−0.4 (−8.3, 2.9)	0.338	0.27	0.8 (−1.4, 6.2)	0.216
Aggressive problems	6.9 (7.8)	3.9 (4.7)	0.64	8.8 (8.9)	4.1 (5.4)	0.91	0.1 (−11.7, 14.4)	0.836	0.77	0.2 (−7.4, 10.3)	0.747
Internalising problems	8.1 (5.7)	7.5 (7.0)	0.13	4.0 (2.5)	4.5 (3.9)	−0.21	0.2 (−8.8, 15.3)	0.585	0.16	−0.3 (−10.4, 6.0)	0.582
Externalising problems	8.3 (9.0)	5.1 (5.7)	0.60	11.8 (13.4)	5.3 (6.4)	0.87	−0.0 (−16.9, 16.0)	0.961	0.73	0.4 (−7.8, 14.6)	0.540
Total problems	25.6 (22.2)	25.9 (21.2)	−0.02	23.6 (22.3)	16.5 (12.8)	0.55	−0.1 (−44.4, 36.4)	0.842	0.28	0.2 (−22.3, 32.5)	0.709
SPAI-C-P^b^											
Assertiveness/General conversation (AGC)	3.9 (3.0)	3.8 (3.2)	0.08	2.7 (2.1)	2.6 (2.4)	0.05	0.1 (−5.3, 7.2)	0.762	0.14	−0.2 (−4.9, 3.6)	0.767
Traditional social encounters (TSE)	1.4 (1.6)	0.9 (1.5)	0.40	1.6 (2.4)	0.5 (1.0)	0.85	0.0 (−3.2, 3.2)	0.984	0.67	0.3 (−1.7, 2.7)	0.643
Public performance (PP)	4.6 (4.5)	4.7 (3.0)	−0.04	4.5 (3.6)	2.2 (2.4)	1.05	−0.3 (−8.8, 4.7)	0.543	0.45	0.6 (−2.3, 2.3)	0.328
Total	12.6 (8.5)	10.2 (8.1)	0.41	9.4 (8.7)	5.9 (5.4)	0.69	0.1 (−13.7, 18.8)	0.757	0.60	0.0 (−10.7, 11.3)	0.956
EAS^c^											
Emotionality	3.2 (1.2)	2.6 (0.9)	0.79	2.8 (1.1)	2.4 (0.8)	0.57	0.4 (−1.1, 2.9)	0.356	0.76	−0.3 (−1.7, 1.0)	0.620
Activity	2.7 (0.8)	3.1 (0.7)	−0.77	3.3 (0.9)	3.0 (0.7)	0.47	0.6 (−2.5, 0.4)	0.153	−0.25	0.6 (−0.4, 1.5)	0.259
Shyness	2.1 (0.7)	2.4 (0.8)	−0.50	2.0 (1.0)	2.0 (0.9)	0.06	−0.3 (−2.4, 1.0)	0.437	−0.18	0.5 (−0.7, 1.6)	0.416
Sociability	3.5 (0.6)	3.3 (1.0)	0.33	3.7 (0.6)	3.3 (1.0)	0.63	0.1 (−1.3, 1.6)	0.831	0.45	−0.1 (−1.0, 0.8)	0.822
SASC-R^d^											
Fear of negative evaluation (FNE)	16.5 (6.9)	14.2 (5.1)	0.53	13.4 (5.6)	13.0 (5.5)	0.10	0.4 (−6.7, 17.1)	0.384	0.36	−0.5 (−11.2, 5.0)	0.445
Social avoidance and distress (SAD) New	16.2 (4.3)	14.0 (3.9)	0.74	14.4 (5.9)	11.6 (3.4)	0.81	0.4 (−4.2, 11.6)	0.348	0.82	−0.3 (−6.7, 3.9)	0.599
Social avoidance and distress (SAD) General	6.2 (2.6)	7.4 (2.9)	−0.61	5.4 (3.1)	5.8 (1.6)	−0.21	−0.3 (−6.9, 3.2)	0.453	−0.41	0.3 (−2.6, 4.2)	0.641
Total	38.8 (12.5)	35.6 (9.3)	0.42	33.2 (14.0)	30.4 (8.5)	0.34	0.3 (−13.5, 27.4)	0.495	0.41	−0.3 (−17.5, 10.2)	0.596
Self-Perception Profile for Children											
Total school competence	3.3 (0.5)	3.1 (0.6)	0.41	3.3 (0.2)	3.3 (0.4)	0.01	0.3 (−0.7, 1.5)	0.485	0.25	−0.3 (−0.9, 0.5)	0.596

Regression coefficients (β) with 95% confidence intervals (CI lower, CI upper), *p* values for the main effect of CAH, interaction between CAH and SEX and effect size (Cohen’s *d*) adjusted for parental education and country of origin are listed for all findings. Significant *p* values are marked in bold

^a^ Child Behaviour Checklist (CBCL)

^b^ Social Phobia and Anxiety Inventory for Children—Parent Report (SPAI-C-P)

^c^ Emotionality–Activity–Sociability–Shyness Temperament Survey for children (EAS)

^d^ Social Anxiety Scale for Children Revised (SASC-R)

### Parent-completed questionnaires

There were no significant differences between the groups or a significant interaction between sex and diagnostic status in the parental ratings of children’s problems on the scales measuring adaptive functioning (CBCL total competence score). In addition, we found no significant differences in behavioural problems when the CBCL subscales were grouped on the level of two broad groupings (Internalising and Externalising problems). Finally, there were no significant differences between the groups on the Total problem score (all *p*s > 0.05, Table 2).

However, the CAH group was significantly different from the controls on the CBCL subscale Social problems (*β* = 0.60, *t*(105) = 2.05, *p* = 0.042, *d* = 0.26) (Table 2). We also found a significant interaction between CAH and sex in Social problems (*β* = −0.85, *t*(105) = −2.17, *p* = 0.032) and the post hoc analyses revealed that girls with CAH were scored by their parents to have significantly more problems than control girls (*F*(1,56) = 4.3, *p* = 0.043). In contrast, boys with CAH did not differ significantly from control boys (*F*(1,49) = 3.5, *p* = 0.067).

The analysis of parental estimations of their children’s social anxiety using the three factors of the SPAI-C-P did not reveal any significant differences between the groups.

In the Temperament Survey for children (EAS) none of the four aspects of temperament analysed showed a significant difference between the groups (*p* > 0.05, Table 2).

### Child-completed questionnaires

In the children’s self-reports of social anxiety, as measured by SASC-R, we did not identify any significant difference between the groups (*p* > 0.05, Table 2).

In the comparison of the children and adolescence’s ratings on scholastic competence in the Self-Perception Profile for Children there were no significant differences between the groups (*p* > 0.05, Table 2).

### Effects of prenatal treatment with DEX

In the parental ratings of children’s behavioural problems (CBCL) we found a significant difference between the groups (CAHDEX vs. CAH) for the main effect of DEX on the subscale Withdrawn/Depressed (*β* = 1.10, *t*(40) = 1.90, *p* = 0.014, *d* = 0.40) and a significant interaction between DEX and sex (*β* = −1.41, *t*(40) = −2.4, *p* = 0.014) (Table 3). However, separate post hoc analyses did not reach statistical significance in girls (*F*(1,20) = 1.3, *p* = 0.266) or boys (*F*(1,17) = 1.7, *p* = 0.210).

We also observed a significant interaction between DEX and sex (*β* = 1.43, *t*(48) = 2.55, *p* = 0.015, Table 3) in the Social problems subscale of the CBCL. The post hoc analyses revealed more social problems in CAHDEX-treated boys compared with boys with CAH that had not received prenatal DEX therapy (*F*(1,17) = 11.5, *p* = 0.003).

There were no significant differences between the groups in the analysis of parental estimations of their children’s social anxiety using the three factors of the SPAI-C-P (*p* > 0.05, Table 3).

In the Temperament Survey for children (EAS) none of the four aspects of temperament analysed showed a significant difference between the groups (*p* > 0.05, Table 3).

There were no significant differences between the groups in the children’s self-reports of social anxiety, as measured by SASC-R nor on scholastic competences in the Self-Perception Profile for Children (*p* > 0.05, Table 3).

### Social problems regression analyses

Neither genotype, phenotype nor GC dosage contributed to the prediction (*p* > 0.5).

## Discussion

In the present study we investigated the behavioural outcomes in a group of children and adolescents with CAH who were diagnosed early via neonatal screening programmes for CAH in Sweden (nationwide) and Italy (Emilia-Romagna region). We also describe the behavioural outcomes in a small group of children and adolescents with CAH who were treated prenatally with DEX. To investigate behavioural problems, adaptive functioning and temperament a battery of questionnaires was completed by parents and children and adolescents. The result of a good overall adjustment was expected, as we previously observed normal intelligence, executive functioning, learning and long-term memory in the current Swedish cohort. Indeed, our patient cohort seems to be well adjusted. Such positive adjustment may be attributed in part to close monitoring and access to continuity of care and psychological support.

The only significant group difference in the parental questionnaires was the higher incidence of social problems among CAH children and adolescents compared with the population controls. Moreover, comparing children and adolescents with CAH who were prenatally treated with DEX with children and adolescents with CAH who had not received such treatment, our results indicate more withdrawn-depressed problems in DEX-treated children and adolescents and more social problems in DEX-treated boys, as reported by their parents (CBCL).

The CBCL Social problems subscale assesses socially immature behaviours and difficulties in establishing close relationships with peers, as assessed with such items as ‘Act too young for his or her age’, ‘Clings to adults or too dependent’ and ‘Does not get along with other kids’. Systematic studies focusing on the possible causes of social problems in children with CAH are scarce. Our results may not be surprising given that it is known that children with a chronic medical condition display less prosocial behaviour with peers [51]. A chronic medical disease may have a negative influence on psychosocial development, especially if promoting positive adaptation is challenging for the parents. Evidence from the literature suggests that the parental emotional state and parenting styles impact the child’s developmental trajectory. Parenting an ill child may lead to high parental stress and associated less effective parental strategies [52].

A systematic review found that parental psychological distress in parents of children with Type 1 diabetes mellitus was associated with more problematic behaviour in children [53], although the causal direction of this association is not clear. Thus, our findings underline the importance of screening and the need for counselling and emotional and psychological support for parents with ill children. Finally, further factors associated with social functioning in girls with CAH may be specific issues, such as body image, sexual functioning and self-stigma.

Of note, studies on behavioural outcome in children with CAH are scarce and those that are available have mostly focused on sex-specific issues (mainly in females) based on the hypothesised link between androgen effects and behavioural development. Previous studies investigating CAH in girls reported a higher level of aggression and a preference for masculine, physical sports and behaviours compared with unaffected females [54, 55]. However, in our cohort we did not observe any difference between girls with CAH and control girls in the subscale of CBCL assessing aggressive behaviour. Aggressive behaviour was assessed using a subscale of the CBCL questionnaire containing such items as ‘Argues a lot”, ‘Cruelty’, ‘Bullying or meanness to others’ and ‘Destroys things belonging to his or her family’.

Some studies have also shown a significant increase in anxiety disorders in boys and girls with CAH [56]. In our study the children and adolescents with CAH did not report more social anxiety compared with the control children and adolescents. Likewise, we did not observe any between-group differences on the Scholastic Competence subscale for self-rated scholastic competence. Thus, there was good agreement between the parental ratings and the children’s and adolescents’ self-ratings, indicating a good overall psychological adjustment in children and adolescents with CAH. Medical treatment with HC and rapid initiation of therapy after neonatal screening may be important factors in helping to maintain psychological well-being.

Our study cohort also included a small number of children and adolescents who had been treated prenatally with DEX. The parents of these children and adolescents reported more social problems in males and more withdrawn/depressed problems in girls. However, because the analyses of these data were limited by poor statistical power, we were not able to draw any formal conclusions. Consequently, we suggest the use of multi-source assessment in larger studies.

In conclusion, our results on behavioural outcomes in patients with CAH confirm previous findings of good overall psychological adjustment in boys and girls with CAH [57, 58]. Using parental questionnaires and children’s self-reports, we did not identify any major behavioural problems. Our results indicate, however, more social problems in children and adolescents with CAH, as well as possibly more withdrawn-depressed problems in DEX-treated children/adolescents and more social problems in DEX-treated boys, as reported by their parents (CBCL). These findings highlight the importance of follow-up until adult age and screening of mental well-being. Integrating emotional and psychological support into the overall clinical management of CAH should be an integral part of the care process.

## Abbreviations

CAH: Congenital adrenal hyperplasia
DEX: Dexamethasone
21OHD: 21-Hydroxylase deficiency
GC: Glucocorticoid
GR: Glucocorticoid receptor
SW: Salt-wasting
SV: Simple virilising
HC: Hydrocortisone

## References

[1] Speiser PW, Arlt W, Auchus RJ, Baskin LS, Conway GS, Merke DP, Meyer-Bahlburg HFL, Miller WL, Murad MH, Oberfield SE, White PC (2018). Congenital adrenal hyperplasia due to steroid 21-hydroxylase deficiency: an Endocrine Society clinical practice guideline. J. Clin. Endocrinol. Metab..

[2] Merke DP, Bornstein SR (2005). Congenital adrenal hyperplasia. Lancet.

[3] Wedell A (2011). Molecular genetics of 21-hydroxylase deficiency. Endocr. Dev..

[4] Balsamo A, Baldazzi L, Menabo S, Cicognani A (2010). Impact of molecular genetics on congenital adrenal hyperplasia management. Sex. Dev..

[5] Thilén A, Nordenström A, Hagenfeldt L, von Döbeln U, Guthenberg C, Larsson A (1998). Benefits of neonatal screening for congenital adrenal hyperplasia (21-hydroxylase deficiency) in Sweden. Pediatrics.

[6] Therrell BL (2001). Newborn screening for congenital adrenal hyperplasia. Endocrinol. Metab. Clin. North Am..

[7] Balsamo A, Cacciari E, Piazzi S, Cassio A, Bozza D, Pirazzoli P, Zappulla F (1996). Congenital adrenal hyperplasia: neonatal mass screening compared with clinical diagnosis only in the Emilia-Romagna region of Italy, 1980-1995. Pediatrics.

[8] F. Baronio, R. Ortolano, S. Menabo, A. Cassio, L. Baldazzi, V. Di Natale, G. Tonti, B. Vestrucci, A. Balsamo, 46,XX DSD due to androgen excess in monogenic disorders of steroidogenesis: genetic, biochemical, and clinical features. Int. J. Mol. Sci. **20**(18), (2019). 10.3390/ijms20184605 10.3390/ijms20184605 PMC6769793 31533357

[9] Angold A (2003). Adolescent depression, cortisol and DHEA. Psychol. Med..

[10] van Goozen SH, Fairchild G, Snoek H, Harold GT (2007). The evidence for a neurobiological model of childhood antisocial behavior. Psychol. Bull..

[11] Stone AA, Schwartz JE, Smyth J, Kirschbaum C, Cohen S, Hellhammer D, Grossman S (2001). Individual differences in the diurnal cycle of salivary free cortisol: a replication of flattened cycles for some individuals. Psychoneuroendocrinology.

[12] Stetler C, Dickerson SS, Miller GE (2004). Uncoupling of social zeitgebers and diurnal cortisol secretion in clinical depression. Psychoneuroendocrinology.

[13] McEwen B, Chao H, Spencer R, Brinton R, Macisaac L, Harrelson A (1987). Corticosteroid receptors in brain: relationship of receptors to effects in stress and aging. Ann. N. Y. Acad. Sci..

[14] Colciago A, Casati L, Negri-Cesi P, Celotti F (2015). Learning and memory: steroids and epigenetics. J. Steroid Biochem. Mol. Biol..

[15] Bergamaschi R, Livieri C, Uggetti C, Candeloro E, Egitto MG, Pichiecchio A, Cosi V, Bastianello S (2006). Brain white matter impairment in congenital adrenal hyperplasia. Arch. Neurol..

[16] Mnif MF, Kamoun M, Mnif F, Charfi N, Kallel N, Rekik N, Naceur BB, Fourati H, Daoud E, Mnif Z, Sfar MH, Younes-Mhenni S, Sfar MT, Hachicha M, Abid M (2013). Brain magnetic resonance imaging findings in adult patients with congenital adrenal hyperplasia: increased frequency of white matter impairment and temporal lobe structures dysgenesis. Indian J. Endocrinol. Metab..

[17] Ernst M, Maheu FS, Schroth E, Hardin J, Golan LG, Cameron J, Allen R, Holzer S, Nelson E, Pine DS, Merke DP (2007). Amygdala function in adolescents with congenital adrenal hyperplasia: a model for the study of early steroid abnormalities. Neuropsychologia.

[18] Berenbaum SA, Beltz AM, Bryk K, McHale S (2018). Gendered peer involvement in girls with congenital adrenal hyperplasia: effects of prenatal androgens, gendered activities, and gender cognitions. Arch. Sex. Behav..

[19] Nordenstrom A, Servin A, Bohlin G, Larsson A, Wedell A (2002). Sex-typed toy play behavior correlates with the degree of prenatal androgen exposure assessed by CYP21 genotype in girls with congenital adrenal hyperplasia. J. Clin. Endocrinol. Metab..

[20] Pasterski V, Hindmarsh P, Geffner M, Brook C, Brain C, Hines M (2007). Increased aggression and activity level in 3- to 11-year-old girls with congenital adrenal hyperplasia (CAH). Hormones Behav..

[21] Pasterski V, Zucker KJ, Hindmarsh PC, Hughes IA, Acerini C, Spencer D, Neufeld S, Hines M (2015). Increased cross-gender identification independent of gender role behavior in girls with congenital adrenal hyperplasia: results from a standardized assessment of 4- to 11-year-old children. Arch. Sex. Behav..

[22] Engberg H, Butwicka A, Nordenstrom A, Hirschberg AL, Falhammar H, Lichtenstein P, Nordenskjold A, Frisen L, Landen M (2015). Congenital adrenal hyperplasia and risk for psychiatric disorders in girls and women born between 1915 and 2010: a total population study. Psychoneuroendocrinology.

[23] Falhammar H, Butwicka A, Landen M, Lichtenstein P, Nordenskjold A, Nordenstrom A, Frisen L (2014). Increased psychiatric morbidity in men with congenital adrenal hyperplasia due to 21-hydroxylase deficiency. J. Clin. Endocrinol. Metab..

[24] Falhammar H, Frisen L, Norrby C, Hirschberg AL, Almqvist C, Nordenskjold A, Nordenstrom A (2014). Increased mortality in patients with congenital adrenal hyperplasia due to 21-hydroxylase deficiency. J. Clin. Endocrinol. Metab..

[25] Sonino N, Fava GA (1998). Psychosomatic aspects of Cushing’s disease. Psychother. Psychosom..

[26] Loosen PT, Chambliss B, DeBold CR, Shelton R, Orth DN (1992). Psychiatric phenomenology in Cushing’s disease. Pharmacopsychiatry.

[27] Devoe DJ, Miller WL, Conte FA, Kaplan SL, Grumbach MM, Rosenthal SM, Wilson CB, Gitelman SE (1997). Long-term outcome in children and adolescents after transsphenoidal surgery for Cushing’s disease. J. Clin. Endocrinol. Metab..

[28] Kelly WF, Kelly MJ, Faragher B (1996). A prospective study of psychiatric and psychological aspects of Cushing’s syndrome. Clin. Endocrinol..

[29] Lajic S, Nordenstrom A, Hirvikoski T (2008). Long-term outcome of prenatal treatment of congenital adrenal hyperplasia. Endocr. Dev..

[30] Miller WL (2015). Fetal endocrine therapy for congenital adrenal hyperplasia should not be done. Best Pract. Res. Clin. Endocrinol. Metab..

[31] Lajic S, Karlsson L, Nordenstrom A (2018). Prenatal treatment of congenital adrenal hyperplasia: long-term effects of excess glucocorticoid exposure. Horm. Res. Paediatr..

[32] Hirvikoski T, Nordenstrom A, Lindholm T, Lindblad F, Ritzen EM, Wedell A, Lajic S (2007). Cognitive functions in children at risk for congenital adrenal hyperplasia treated prenatally with dexamethasone. J. Clin. Endocrinol. Metab..

[33] T. Hirvikoski, T. Lindholm, A. Nordenstrom, A.L. Nordstrom, S. Lajic, High self-perceived stress and many stressors, but normal diurnal cortisol rhythm, in adults with ADHD (attention-deficit/hyperactivity disorder). Hormones Behav. 55(3), 418–424 (2009). S0018-506×(08)00339-5 [pii] 10.1016/j.yhbeh.2008.12.004 10.1016/j.yhbeh.2008.12.004 19162030

[34] Wallensteen L, Karlsson L, Messina V, Gezelius A, Sandberg MT, Nordenstrom A, Hirvikoski T, Lajic S (2018). Evaluation of behavioral problems after prenatal dexamethasone treatment in Swedish children and adolescents at risk of congenital adrenal hyperplasia. Horm. Behav..

[35] Holmbeck GN, Johnson SZ, Wills KE, McKernon W, Rose B, Erklin S, Kemper T (2002). Observed and perceived parental overprotection in relation to psychosocial adjustment in preadolescents with a physical disability: the mediational role of behavioral autonomy. J. Consult Clin. Psychol..

[36] Stein D, Williamson DE, Birmaher B, Brent DA, Kaufman J, Dahl RE, Perel JM, Ryan ND (2000). Parent-child bonding and family functioning in depressed children and children at high risk and low risk for future depression. J. Am. Acad. Child Adolesc. Psychiatry.

[37] Spada MM, Caselli G, Manfredi C, Rebecchi D, Rovetto F, Ruggiero GM, Nikcevic AV, Sassaroli S (2012). Parental overprotection and metacognitions as predictors of worry and anxiety. Behav. Cogn. Psychother..

[38] Frisen L, Nordenstrom A, Falhammar H, Filipsson H, Holmdahl G, Janson PO, Thoren M, Hagenfeldt K, Moller A, Nordenskjold A (2009). Gender role behavior, sexuality, and psychosocial adaptation in women with congenital adrenal hyperplasia due to CYP21A2 deficiency. J. Clin. Endocrinol. Metab..

[39] Wallensteen L, Zimmermann M, Thomsen Sandberg M, Gezelius A, Nordenstrom A, Hirvikoski T, Lajic S (2016). Sex-dimorphic effects of prenatal treatment with dexamethasone. J. Clin. Endocrinol. Metab..

[40] Karlsson L, Gezelius A, Nordenstrom A, Hirvikoski T, Lajic S (2017). Cognitive impairment in adolescents and adults with congenital adrenal hyperplasia. Clin. Endocrinol..

[41] Karlsson L, Nordenstrom A, Hirvikoski T, Lajic S (2018). Prenatal dexamethasone treatment in the context of at risk CAH pregnancies: long-term behavioral and cognitive outcome. Psychoneuroendocrinology.

[42] Wikland KA, Luo ZC, Niklasson A, Karlberg J (2002). Swedish population-based longitudinal reference values from birth to 18 years of age for height, weight and head circumference. Acta Paediatrica.

[43] Bertino E, Spada E, Occhi L, Coscia A, Giuliani F, Gagliardi L, Gilli G, Bona G, Fabris C, De Curtis M, Milani S (2010). Neonatal anthropometric charts: the Italian neonatal study compared with other European studies. J. Pediatr. Gastroenterol. Nutr..

[44] Achenbach TM (1991). Manual for the Child Behavior Checklist/4-18 and 1991 Profile.

[45] Beidel DC, Turner SM, Morris TL (1995). A new inventory to assess social phobia in children: The Social Phobia and Anxiety Inventory for Children. Psychol. Assess..

[46] Higa CK, Fernandez SN, Nakamura BJ, Chorpita BF, Daleiden EL (2006). Parental assessment of childhood social phobia: psychometric properties of the social phobia and anxiety inventory for children-parent report. J. Clin. Child Adolesc. Psychol..

[47] Buss AH, Plomin R (1984). Temperament: Early Developing Personality Traits..

[48] La Greca AM, Dandes SK, Wick P, Shaw K, Stone WL (1988). Development of the Social Anxiety Scale for Children: reliability and concurrent validity. J. Clin. Child Psychol..

[49] La Greca AM, Stone WL (1993). Social Anxiety Scale for Children-revised: factor structure and concurrent validity. J. Clin. Child Psychol..

[50] S. Harter, The Self-perception Profile for Children: Revision of the Perceived Competence Scale for Children (University of Denver, Colorado, USA, 1985)

[51] Meijer SA, Sinnema G, Bijstra JO, Mellenbergh GJ, Wolters WH (2000). Social functioning in children with a chronic illness. J. Child Psychol. Psychiatry.

[52] Williams HL, Cullen LA, Barlow JH (2005). The psychological well-being and self-efficacy of carers of children with disabilities following attendance on a simple massage training and support programme: a 12-month comparison study of adherers and non-adherers. Complement Ther. Med..

[53] Whittemore R, Jaser S, Chao A, Jang M, Grey M (2012). Psychological experience of parents of children with type 1 diabetes: a systematic mixed-studies review. Diabetes Educ..

[54] Berenbaum SA, Resnick SM (1997). Early androgen effects on aggression in children and adults with congenital adrenal hyperplasia. Psychoneuroendocrinology.

[55] Berenbaum SA (1999). Effects of early androgens on sex-typed activities and interests in adolescents with congenital adrenal hyperplasia. Hormones Behav..

[56] Mueller SC, Ng P, Sinaii N, Leschek EW, Green-Golan L, VanRyzin C, Ernst M, Merke DP (2010). Psychiatric characterization of children with genetic causes of hyperandrogenism. Eur. J. Endocrinol..

[57] Berenbaum SA, Korman Bryk K, Duck SC, Resnick SM (2004). Psychological adjustment in children and adults with congenital adrenal hyperplasia. J. Pediatrics.

[58] Berenbaum SA, Bryk KK, Duck SC (2010). Normal intelligence in female and male patients with congenital adrenal hyperplasia. Int J. Pediatr. Endocrinol..

